# Modeling and Characterization of Surface Discharges in Insulating Material for Spacers: Electrode Shape, Discharge Mode, and Revision of the Creepage Concept

**DOI:** 10.3390/ma16030989

**Published:** 2023-01-20

**Authors:** Debasish Nath, Qichen Yang, Giancarlo Montanari, Weijun Yin, Han Xiong, Karim Younsi

**Affiliations:** 1 CAPS, Florida State University, Tallahassee, FL 32310, USA; 2 GE Research, Niskayuna, NY 12309, USA

**Keywords:** partial discharge, gas discharge, space charge, electric field, spacer, insulators, triple point

## Abstract

In the design of MV AC and DC spacers, the predominant factors are surface and interface conditions. Design is generally carried out on specifications and standards which are based on long-term experience and lab testing. However, the diffusion of power electronics with a trend to increase electric field, switching frequency, and rise time to achieve higher power density calls for an innovative, global approach to optimized insulation system design. A new methodology, based on field simulation, discharge modeling, and partial discharge inception measurements, called the three-leg approach, can form the basis to optimize insulation design for any type of supply voltage waveform. This paper focuses on the influence of the type of electrode on the inception and phenomenology of surface discharges and, as a consequence, on the interpretation of the results used for application of the three-leg approach. It is demonstrated that a typical electrode system used for insulating material testing can generate both gas and surface discharges at the triple point, when the electrodes have a smooth profile that is used to avoid corona or flashover. Hence, testing partial discharge may not provide a straightforward indication of the surface discharge inception and, thus, be partially misleading for insulation design. Another takeover is that such analysis must benefit from PD testing tools endowed with analytics able to provide automatic identification of the type of defect generating PD, i.e., internal, surface, and corona, since design and remedy actions can be taken, and adequate insulating materials developed, only knowing the type of source generating PD. Hence, testing partial discharge may not provide a straightforward indication of surface discharge inception and, thus, be partially misleading for insulation design. In addition to the importance of the three-leg approach to favor reliable and optimized design of insulation systems, there is a clear need to have a PD testing tool endowed with analytics. It should preferably be able to provide automatic identification of the type of defect generating PD, i.e., internal, surface, and corona.

## 1. Introduction

New supply and operating environments, such as in transportation electrification, may have a huge impact on insulation system reliability. The design of insulation systems, such as MV and HV spacers, is often based on experience coming from decades of on-field installation, but this approach may no longer cope with insulation reliability specifications [1,2,3]. Indeed, power electronics can reach such large electrothermal stress levels and such different voltage waveforms from sinusoidal AC that they cause accelerated intrinsic and extrinsic aging, unimagined in conventional insulation system design that has been based on long-term experience under sinusoidal AC voltage.

Hence, insulation thickness, contact/electrode shape, bulk design field, surface clearance, and creepage might need to change drastically with voltage waveform, magnitude, and environmental conditions, as well as pressure and contamination.

According to the new design criterion introduced in recent papers, which we call the “three-leg approach” [4,5], the way to face the challenge of reaching a design that can provide the specified reliability and life must stem from the modeling of extrinsic and intrinsic aging mechanism, such as surface discharges, the knowledge of stress profiles (e.g., electric field distribution in the insulation system), and the validation based on diagnostic property and extrinsic aging quantity measurements, e.g., the partial discharge voltage (PDIV) [4,5,6]. In this way, the risk of inception of extrinsic aging phenomena that can be destructive from insulation can be accounted for at the design stage, together with the estimation of design stresses associated with intrinsic aging [6,7,8,9].

An Important note about PD measurements is that they cannot be carried out generically as described in [10]. Indeed, PD detection must be associated with a level of analytics which must be able to recognize PD from noise both in DC and AC (sinusoidal and modulated), and to identify the type of source generating PD (internal cavities, surface, or corona discharges), because PD harmfulness evaluation, extrinsic aging rate, and relevant remedy actions depend on this identification [11,12,13]. In other words, measuring PD without understanding the phenomena that generate them can be misleading for the design of an insulation system, since design and remedy actions change depending on PD typology. In particular, to achieve a PD-free design, the risk of PD inception or of the presence of PD during operation must be addressed to surface or internal (gas) discharges [14,15].

Focusing on surface insulation subsystem design, laboratory tests are often carried out for each candidate material, using electrodes connected to high voltage and ground, placed on a flat specimen. Once PDs are incepted, the PDIV for the test object is established and the distance between electrodes is varied in order to prove that at the nominal operating voltage discharges would not be incepted. Concepts such as creepage and clearance have been developed to design the surface subsystem of an insulation, as described in Section 2, which may not work properly for a reliable design when the electric field is not uniform [4,5,16]. Depending on the electrode shape, however, PD may incept not only due to the tangential field to the insulation surface, but also due to discharges in the air (gas) gap between electrode and specimen surface. Having models that can describe both discharge phenomena, therefore, becomes a must to go from experimental lab results to the design of an insulation system, addressing its reliability and life prediction. Such models are dealt with in Section 3, while the application of this speculative background to characterize surface discharges in specimens of a target material for spacer insulation is addressed in Section 4. The manuscript ends with a discussion and conclusions.

## 2. Creepage, Clearance, Field Distribution, and Surface Discharge Inception

**Figure 1:**
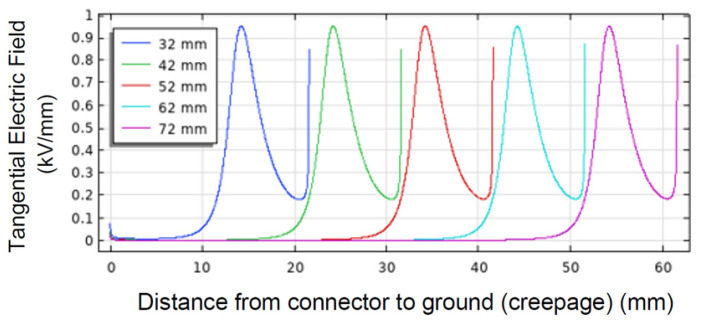
Field profile on PCB surface as a function of creepage distance. The maximum value does not change noticeably going from creepage of 32 mm to 72 mm.

A fundamental design feature for MV/HV spacers is to optimize surface insulation system design, in which creepage and clearance are paramount quantities, specified in various standards [16]. In particular, they are meant to reduce the risk of macroscopic discharges that can instantly break the insulation system. However, it is likely that macroscopic surface discharges are preceded by surface partial discharges, PD, originated from triple points (electrode, surface, and surrounding medium), contamination (droplets, salt, and impurity clusters), or, in general, large field gradients. If the voltage at which surface PDs are incepted is lower than the nominal voltage recommended for insulation system operation (based on creepage criteria), the insulation will not undergo macroscopic discharges, but it will degrade rapidly at the PD location (extrinsic accelerated aging), resulting in premature failure (thus, lower-than-specified reliability). It is noteworthy that the occurrence of PD could not be hampered using the concept of creepage. As an example, Figure 1 shows how the maximum field on a PCB surface (from [4]) does not vary noticeably even when doubling the creepage. Hence, creepage and PD inception field/voltage must be considered in *AND* logic when designing surface insulation sub-system.

Therefore, calculating insulator surface field the PD inception field through appropriate modeling (described in the next section), followed by validating the surface design maximum field and voltage by PDIV measurements, seems to be the most appropriate approach to establish the basis for an optimized insulator design. This is, indeed, an application of the three-leg approach, which has to go side by side with a reliable design of bulk insulation. This will hold for AC sinusoidal, modulated, and DC supply, including voltage and load transients. It is noteworthy that the PD inception condition for both surface and gas (including internal defect) discharges is a function of local field (that will change with the type of voltage supply), conductive part shapes, and material parameters (that will not).

## 3. Discharge Modeling and PDIV

The basic information to be achieved is partial discharge inception voltage, PDIV, with the type of materials and the geometry expected for the insulation system, in order to have confidence that the insulation system design will allow reliable operation for the specified life at nominal voltage (and temperature).

The presence of PD prompts extrinsic aging which will overrule any design (generally based on stresses related to intrinsic aging and related life models [17]). Conventionally, the design field providing specified life at the requested failure probability is determined by accelerated life tests and does not generally account for the possible inception of PD and any other extrinsic aging mechanism (space charge in DC [18], hotspots, etc.). However, as mentioned above, the critical stress environment that modern electrical assets are going to suffer must drive an innovative design ensuring that extrinsic aging mechanisms are not incepted in an insulation system after commissioning and for the whole expected life. Modeling the inception level of such mechanisms, as the inception field for PD, is essential for an optimized, safe, and resilient design.

In general, laboratory tests are carried out, before designing the whole insulation system with its final geometry, to investigate surface and internal discharge inception in specimens of candidate materials.

The results of these tests can be generalized to any geometry or type of defect if discharge models are developed. They must comprehend gas discharges, i.e., those occurring in cavities, delamination, and interfaces, driven by the field orthogonal to the defect, and surface discharges, driven by the field tangential to the insulation surface.

### 3.1. Modeling Gas Discharge

A rough (deterministic) estimation of the PD inception field, *E_ig_*, in the gas of cavities embedded in the insulation bulk (thus mostly sensitive to orthogonal field) is expressed as follows [19]:

(1) $$E_{ig}=25.2p\left(1+\frac{B}{\sqrt{ph}}\right),$$

valid for air at pressure *p* and for a spherical cavity of diameter *h* filled with gas (this expression holds approximately also for cylindrical cavities, considering cavity height *h*). In this expression, *B* can vary from 5.4 to 8.6 depending on cavity size and shape [19,20,21].

### 3.2. Modeling Surface Discharge

With regard to surface discharges, the derivation can follow the same structure as in [20], but the assumption of uniform electric field in the gas space may not hold any more on the insulation surface. Depending on the electrode/metallic part shape and location, the electric field profile may show a significant gradient, as in Figure 1. Thus, solving the critical avalanche condition integral can be cumbersome, and it would depend on all possible combinations of electrodes, materials, and creepage. A new approach was presented in [22], where an approximate solution consists of singling out a surface/volume layer in which the field is maximum and almost uniform, addressing the field divergency to a shape factor *k_s_*. In this way, the surface discharge model is derived from the gas discharge one, with the additional presence of *k_s_* which becomes equal to 1 when the field is uniform (thus, the design surface length approaches the creepage concept):

(2) Eis=Epcr.p.1+Bpksl1βr,

(3) With B=kcrC1βrEpcr,

where ${\left(\frac{E}{p}\right)}_{cr}$ is the pressure reduced critical field, $k_{cr}$ is the logarithm of the critical number of electrons that has to accumulate in the avalanche head to make it self-propagating, C is a quantity proportional to the ionization coefficient, $\beta_{r}$ is a parameter which controls the steepness of the ionization coefficient, when the background electric field exceeds the critical value, and l is the distance between HV conductor (triple point) and ground (creepage).

In addition to introducing $k_{s}$, different parameter values must be taken with respect to gas discharges, considering that the interface between insulating material and gas will influence the ionization and streamer processes [4,5,21,22].

Recent experiments have shown that, for a clean surface, the value of ${\left(\frac{E}{p}\right)}_{cr}$ can be lower than that for gas discharge, Equation (1), i.e., ~8, then $k_{cr}\;$≥ 9 and $\beta_{r}$ = 2 as for gas discharges, but C could be higher than in Equation (1), e.g., $C=7.6\times 10^{-3}$ (it can be speculated that the contribution of the surface is to make easier gas ionization) [22].

For air and under uniform surface tangential field, Equation (2) can be written as

(4) $$E_{is}=8p\left(1+\frac{4.3}{{\left(pk_{s}l\right)}^{0.5}}\right).$$

Approximate expressions for $k_{s}$, based on the extent of field gradient at the triple point and, thus, on the effective streamer length, are given (based on profiles in Figure 1, where the field profile is not monotonic) below [22].

(5) $$k_{s}=\frac{l{\left(0.95E_{max}\right)}^{+}-l{\left(0.95E_{max}\right)}^{-}}{d},$$

or, when the field is monotonically decreasing,

(6) $$k_{s}=\frac{l\left(0.95E_{max}\right)-l\left(0.8E_{max}\right)}{d},$$

where $E_{max}$ *is* the maximum value of tangential surface field as obtained from electric field profile calculation.

### 3.3. Partial Discharge Inception Voltage and PD measurements

The partial discharge inception voltage corresponding to *E_is_* or *E_ig_* can be calculated analytically when dealing with internal discharges and a known defect (shape, size, and location; see, e.g., [23,24] for cables).

When, however, surface discharges are investigated, the straightforward solution to estimate PDIV based on *E_is_* is to carry out simulations at increasing voltage until the maximum field (or, better, that defined by *k_s_*) is reached.

With regard to PD measurements, they can be effective for design purposes only if the type of defect generating PD, i.e., whether internal or surface, can be provided by the analytics associated to the PD detector software. As mentioned, any design or remedial action that can be taken on the basis of PD assessment must be driven by the type and harmfulness of PD. Therefore, innovative algorithms, such as those proposed in [11,12], which are able to automatically separate and recognize noise and PD, as well as identify the source generating PD, would be needed to infer PD phenomenology.

**Figure 2:**
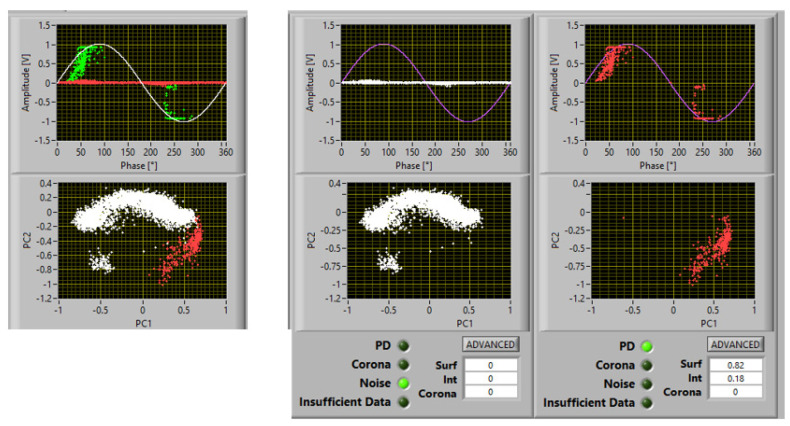
Example of PD acquisition. Recorded phenomena are separated (PCA map), noise is recognized and rejected, and PD source typology is identified. Test voltage: 1.1 PDIV. There are two subclusters in the PCA map, one recognized as noise and one recognized as PD, with identification of surface discharges with large a likelihood (82%).

As an example, Figure 2 shows a screenshot of PD acquisition according to the innovative approach which allows recorded phenomena to be separated (principal component analysis (PCA) map), noise to be recognized and rejected, and PD source typology to be identified. The figure reports a case of measurements at 1.1 PDIV on a spacer insulating material specimen, with two subclusters in the PCA map, one recognized as noise and one recognized as PD, with identification of surface discharges with a large likelihood (82%).

### 3.4. Application of the Three-Leg Approach

The three-leg approach has the purpose of driving the optimized design of insulation systems with regard to their reliability, life, and dimensions/weight, taking into account the risk of generating extrinsic aging phenomena. The three-legs are as follows:

A- Stress profile calculation (simulation) for electrothermal–mechanical stresses.
B- Intrinsic aging and discharge modeling, in order to estimate (in the case of surface insulation design) the inception of surface PD (Equations (1) and (2)). PDIV is estimated through leg A.
C- Validation of the PDIV estimation and inherent discharge model through PDIV measurements, which is related to the inception of extrinsic aging mechanisms under electrothermal–mechanical stresses expected during operating conditions.

Referring to surface discharges, the criterion for design is to stay below the surface PDIV; thus, the surface (tangential) field must be lower than the inception value (Equation (4)).

The choice of test electrode shape can affect PDIV measurements, possibly giving rise to misleading results which would not allow, if not analyzed properly, PDIV to be associated effectively with the inception field for surface discharge determined by the model. This is discussed in the next section, referring to specimens of insulating material for aerospace application.

## 4. PDIV Measurements and Surface PD Inception Field Model Validation

### 4.1. Test Assembly and Procedures

**Figure 3:**
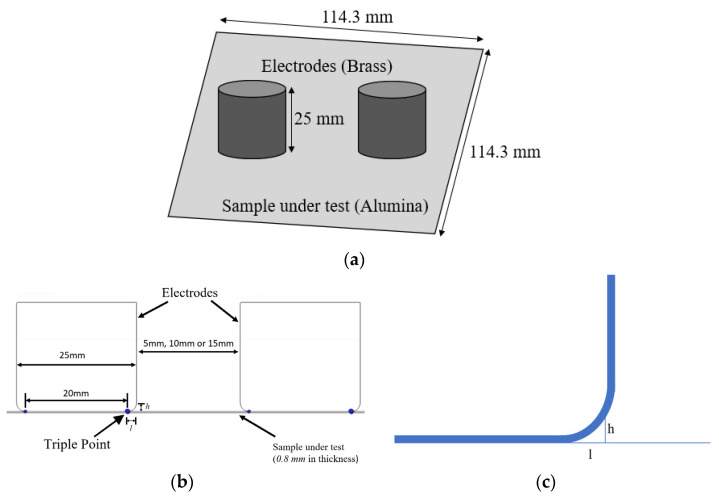
Testing electrodes: (**a**) 3D view of electrode and test sample; (**b**) electrode configuration; (**c**) highlighted electrode contour.

A sketch of the electrode system and tested specimen is displayed in Figure 3, where the electrode contour is highlighted; *l* is the distance along the horizontal direction from the triple point, and *h* is the corresponding orthogonal distance from the sample to the electrode surface.

Specimens were tested where 0.8 mm thick and electrodes were displaced at three different distances, i.e., 5, 10, and 15 mm, on the specimen surface, with one connected to HV, and the other connected to ground. The voltage (AC sinusoidal) was increased stepwise until PDIV was reached, and then further increased to 1.5 PDIV to have more clear evidence of the phenomenon generating PD (identification). Tests were performed at room temperature and atmospheric pressure in a controlled environment.

#### 4.1.1. Calculating the Inception of Internal and Surface Discharges

Two different discharge processes can be incepted. The first is the surface discharge along the horizontal direction, driven by tangential field over the surface of the sample (along *l*; Figure 3b). The second is the gas discharge in orthogonal direction, between the sample and the electrode (along *h*), which would resemble, from the point of view of phenomenology and physics behind, an internal discharge.

The inception field for each of these processes can be estimated using the models in Equations (2) and (1), respectively.

The theoretical value of the inception field, for discharge to initiate in the gas gap between the conductor surface and specimen, over the height *h*, can be derived using Equation (1), with *B* = 5.8.

With regard to surface discharges, Equation (2) model parameters, as estimated from a series of preliminary tests, were those of Equation (4), with *k_s_* given by Equation (5). To derive *k_s_*, the field profile has to be calculated as in the next subsection.

#### 4.1.2. Electric Field Simulation

**Figure 4:**
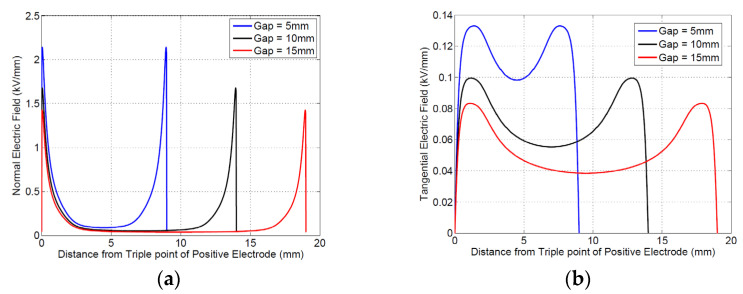
Simulated electric field with electrodes as shown in Figure 3: (**a**) normal field (orthogonal to specimen surface); (**b**) tangential field. The total distance shown along the x-axis is from triple point to triple point, with the triple point of the positive electrode as the origin. The gap (5 mm, 10 mm, or 15 mm) is between the electrode faces. Due to the electrode curvature, the triple point is located at 1.5 mm behind the face of each electrode.

The electric field was simulated (by COMSOL) for an excitation voltage of 1 kV, AC sinusoidal 60 Hz. The normalized fields (scaled with respect to maximum value) are presented in Figure 4, with orthogonal (normal) and tangential components (in directions *h* and *l*, respectively; Figure 3b). The maximum and minimum values of the fields, corresponding to an applied voltage of 1 kV, are summarized in Table 1. Here, the minimum value is that corresponding to the flat portion of the curve, midway through the gap between the electrodes. As can be seen from Figure 4 and Table 1, the maximum value and the difference between maximum and minimum vales do not change significantly with the distance between electrodes, i.e., creepage. This indicates that increasing creepage would not help in significantly increasing the PDIV.

Calculation of *k_s_* can be managed from electric field profiles (nonmonotone) using Equation (5); the values for different electrode distances are provided in Table 2.

#### 4.1.3. Theoretical Inception Field and PDIV Values

**Figure 5:**
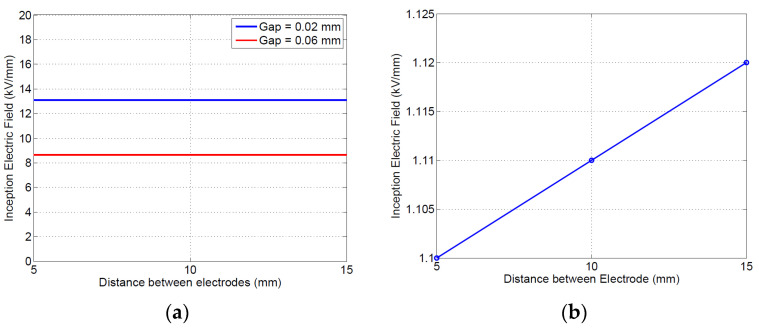
Theoretical inception field for (**a**) internal (gas) discharge, with h = 0.02 and 0.06 mm, and (**b**) surface discharge.

Using Equations (1) and (2), the latter with the value of *k_s_* in Table 2, it is possible to estimate the theoretical inception field for internal (gas) discharge and surface discharge at the triple point. The computed theoretical inception fields are shown in Figure 5. As can be seen, the gas inception field (*h* direction) decreases with distance from the triple point, since the air gap increases, and it does not change significantly with electrode inter-spacing. The surface discharge inception field does not vary significantly with electrode distance either, with the field gradient being the predominant factor (which shows, again, how the common surface creepage design criteria may fail in the presence of uneven surface field distribution).

With the electric fields calculated at an applied voltage of 1 kV, the corresponding fields at any other excitation voltage can be obtained by scaling the fields with the applied voltage (in kV). The simulated electric field can, therefore, be compared with the theoretical inception field, at different applied voltages. The voltage at which the simulated field just exceeds the theoretical inception field is considered as the theoretical partial discharge inception voltage. Either or both the processes described earlier (gas discharge and surface discharge) can potentially be incepted; therefore, the inception for internal and surface discharges must be separately checked because they can impact, in different ways, the generalization of the lab testing to various types of insulation system design.

**Figure 6:**
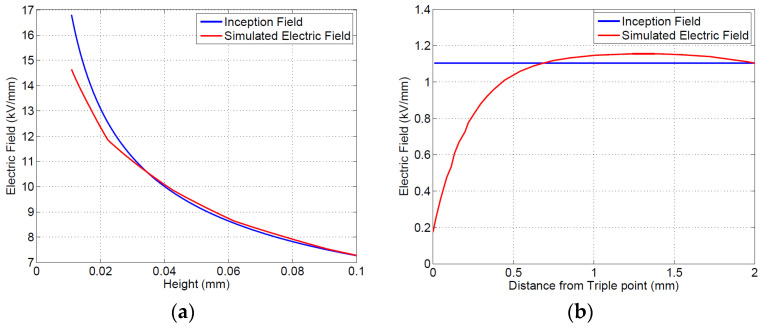
Theoretical inception condition, at electrode separation of 5 mm: (**a**) internal (gas) discharge; (**b**) surface discharge.

Simulation and modeling results on insulating material specimens shown in Figure 6 highlight that both gas and surface discharges may be incepted between 8.6 and 8.4 kV, for an electrode separation of 5 mm (the latter, i.e., surface discharge, seem to be slightly favorite). Upon increasing distance, the inception voltage for surface discharges increases to 13.5 kV, while that for gas discharge increases to 13.2 (see Table 3); thus, the likelihood of gas discharges is still comparable with that of surface discharges for the tested specimens (note that the gas discharge would incept very near to the electrode (Figure 6a), i.e., 40–50 μm, while surface discharge would occur at about 0.6 from the triple point.

The identification of the type of PD provided by the PD software analytics used for these testing, thus, needs to be able to confirm that the PD incepted is indeed surface or gas discharge.

The predicted PDIE and PDIV at the three different electrode separations considered are summarized in Table 3, together with the calculated values of inception field (PDIE is the PD inception field).

### 4.2. Experimental Estimation of PDIV

Table 4 summarizes the experimental and calculated (theoretical) inception voltage values for the tested specimen, the specific electrodes, and the distances between them used for this investigation. Note that the calculated PDIVs for both gap and surface discharge are provided. As can be seen, the theoretical values of PDIV, derived from two of the legs, are validated by the third leg, which means that the proposed discharge models are reasonably accurate to provide design hints for the tested material.

**Figure 7:**
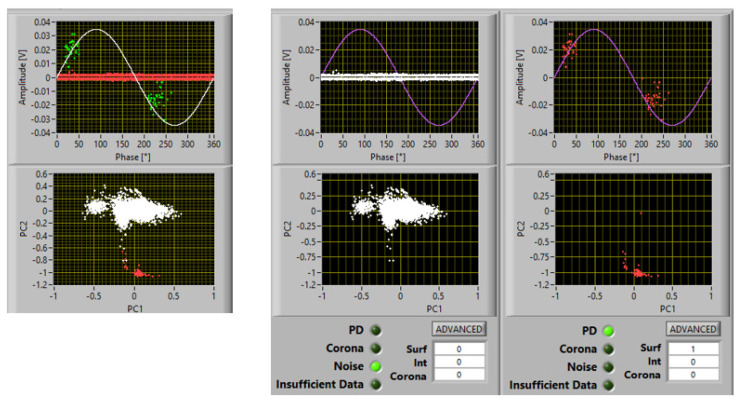
Acquisition and identification of PD activity at 1.1 PDIV, for the tested insulating materials and the electrodes in Figure 3: electrode separation of 5 mm.

**Figure 8:**
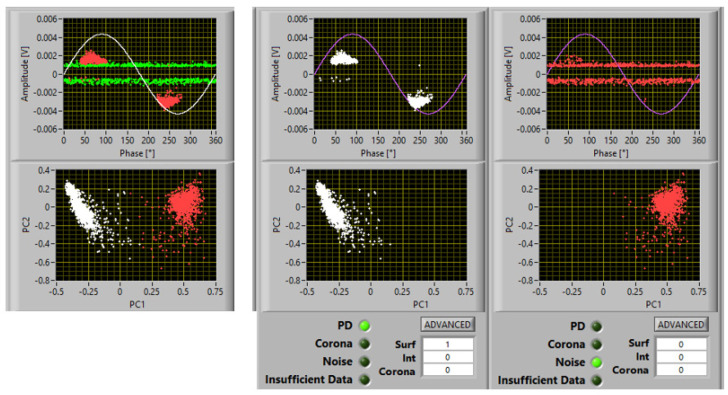
Acquisition and identification of PD activity at 1.1 PDIV, for the tested insulating materials and the electrodes in Figure 3: electrode separation of 10 mm.

**Figure 9:**
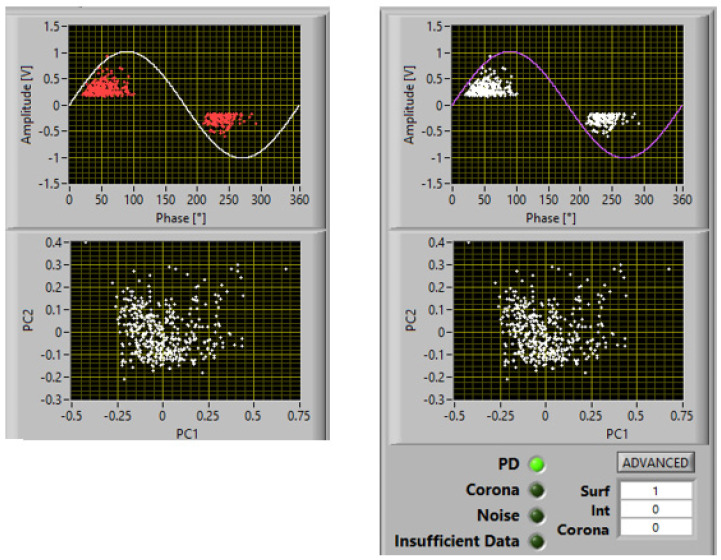
Acquisition and identification of PD activity at 1.1 PDIV, for the tested insulating materials and the electrodes in Figure 3: electrode separation of 15 mm.

Examples of PD acquisition screenshots at 1.1 PDIV are reported in Figure 7, Figure 8 and Figure 9 with 5 mm, 10 mm, and 15 mm electrode gaps, respectively. To briefly explain the contents of the screenshot, it must be reminded that, as described in [11,12], the innovative, automatic, and unsupervised PD measurement/monitoring algorithm used for this research activity (developed by one of the authors) is structured according to the SRI acronym: separation of various types of acquired pulses, recognition of those relevant to PD and to noise, derivation of sub-patterns corresponding to the separated set of pulses, and identification of the type of defect generating PD. This last step (achieved by means of AI algorithms) is associated with PD harmfulness; hence, the chosen PD categories are internal (or gas) discharges, surface discharges, and corona discharges (in descending level of harmfulness and, thus, of extrinsic aging rate). Each screenshot provides the global phase-resolved PD (PRPD) pattern, the separation map (based on principal component analysis (PCA)) of pulse features; below, the sub-patterns for each separated type of pulses are shown, together with the relevant identification (internal or surface corona), along with the associated likelihood (1 means sure identification).

It can be seen that the automatic identification algorithm Indicated a likelihood of 1 for surface discharges; therefore, that it can be speculated that the inception mechanism is initiated by tangential field, with spreading of discharge activity over the specimen surface increasing with voltage. According to electric field profile simulation, Figure 6b, PD would be triggered at a distance of about 600 μm from the triple point. Optical observations indeed showed that some PD erosion started a few hundred microns from the triple point after some minutes under 1.1 PDIV.

It Is noteworthy that, above PDIV, both gas and surface discharge phenomena could be incepted; thus, an identification that can provide the likelihood of the type of PD being active can further help in understanding PD phenomenology, as well as in insulation systems where both internal and surface discharge can coexist.

## 5. Conclusions

A first takeaway from the comparison of PD measurements, inception model, and electric field simulation, i.e., the three legs of the new PD-free design approach, is that the discharge models previously derived and here used for the tested specimens using the electrodes as in Figure 3 seem to work to describe PD inception phenomenology. Therefore, this methodology can help in understanding which type of defect is generating PDs and if they are mostly driven by the orthogonal or tangential field, which is also related to the relevant extrinsic aging rate. Indeed, the PDIV estimated by the models (Equations (1) and (2)), at different separation distances between the electrodes, closely matches the PDIV obtained through measurements and analysis of PD pulses.

These results would allow the PD inception field for insulation components to be estimated and the design field to be derived, through electric field calculations, for any insulation structure made of the tested insulating material. Validation by enhanced PD measurement tools, able to distinguish among the types of PD phenomena, would case-by-case confirm the quality of the design.

This paper, in conclusion, shows how the application of the so-called three-leg approach on simple insulation systems where it is possible to generate surface discharges under controlled conditions, measure partial discharges (PD), and simulate the field distribution can be proficient for material property characterization and insulation system design. The result is a global model able to provide an estimate of the partial discharge inception field, as well as to ensure life and reliability requested by specifications, thereby optimizing the design of the surface insulation for spacers and terminations. This method can constitute an add up to creepage-based design, since it can be speculated that surface partial discharge activity may be detrimental to insulation reliability even if small and localized.

## Figures and Tables

**Table 1:** Maximum and minimum values of electric field (tangential and normal) at an applied voltage of 1 kV.

Electric Field (kV/mm)	Separation Distance between Electrodes					
	5 mm		10 mm		15 mm	
	Etan	Enorm	Etan	Enorm	Etan	Enorm
Minimum	0.1	0.1	0.05	0.05	0.04	0.04
Maximum	0.13	2.1	0.1	1.7	0.08	1.4

**Table 2:** Values of *k*_s_, calculated from the simulated tangential field profile, from Equation (5).

5 mm	10 mm	15 mm
0.28	0.13	0.09

**Table 3:** Theoretical inception fields (PDIE) and inception voltage (PDIV) for surface and gas discharge.

Gap between Electrodes (mm)	Tested Insulating Material			
	Surface Discharge		Gas Discharge	
	Calculated PDIE (kV/mm)	Calculated PDIV (kV)	Calculated PDIE (kV/mm)	Calculated PDIV (kV)
5	1.10	8.4	9.6	8.6
10	1.11	11.2	9.3	11.0
15	1.12	13.5	10.7	13.2

**Table 4:** Experimental PDIV (mean measured values) at different electrode separations, compared to theoretical values.

Gap between Electrodes (mm)	Tested Insulating Material		
	Calculated PDIV (kV) Surface Discharge	Calculated PDIV (kV) Gas Discharge	Measured PDIV (kV)
5	8.4	8.6	8.9
10	11.2	11.0	12.2
15	13.5	13.2	13.3

## Data Availability

Not applicable.
